# Protective humoral and CD4^+^ T cellular immune responses of *Staphylococcus aureus* vaccine MntC in a murine peritonitis model

**DOI:** 10.1038/s41598-018-22044-y

**Published:** 2018-02-26

**Authors:** Wei Yu, Di Yao, Simiao Yu, Xintong Wang, Xiaoting Li, Mengyao Wang, Shuo Liu, Zhenyue Feng, Xiaoting Chen, Wanyu Li, Lizi Wang, Wei Liu, Jinzhu Ma, Liquan Yu, Chunyu Tong, Baifen Song, Yudong Cui

**Affiliations:** College of Life Science and Technology, Heilongjiang Bayi Agricultural University, Daqing, Heilongjiang Province 163319 China

## Abstract

*Staphylococcus aureus* can cause different types of diseases from mild skin infections to life-threatening sepsis worldwide. Owing to the emergence and transmission of multidrug-resistant strains, developing an impactful immunotherapy especially vaccine control approach against *S*. *aureus* infections is increasingly encouraged and supported. *S*. *aureus* manganese transport protein C (MntC), which is a highly-conserved cell surface protein, can elicit protective immunity against *S*. *aureus* and *Staphylococcus epidermidis*. In this study, we evaluated the humoral immune response and CD4^+^ T cell-mediated immune responses in a mouse peritonitis model. The results showed that MntC-specific antibodies conferred an essential protection for mice to reduce invasion of *S*. *aureus*, which was corroborated via the opsonophagocytic killing assay and passive immunization experiment in mice, and moreover MntC-induced Th17 played a remarkable part in preventing *S*. *aureus* infection since the MntC-induced protective immunity decreased after neutralization of IL-17 by antibody *in vivo* and the Th17 adoptive transferred-mice could partly resist *S*. *aureus* challenge. In conclusion, we considered that the MntC-specific antibodies and MntC-specific Th17 cells play cooperative roles in the prevention of *S*. *aureus* infection.

## Introduction

*Staphylococcus aureus* is the most common pathogenic bacterium in humans and animals. It can cause multi-organ infection, pneumonia, pseudomembranous colitis and endocarditis, as well as life-threatening systemic diseases such as septicaemia, sepsis and peritonitis^1,2^. In the past, *S*. *aureus*-caused infection was commonly treated with vancomycin and methicillin antibiotics in clinical practice^3^. However, with the extensive use of antibiotics, many drug-resistant strains have appeared. In the face of the growing drug-resistance of *S*. *aureus*, development of an effective vaccine against this pathogen is particularly important.

Recently researchers have carried out clinical trials of vaccines, including capsular polysaccharide vaccine, subunit vaccine and multivalent capsular polysaccharide with recombinant proteins vaccine. A study of capsular polysaccharide vaccine StaphVAX™, the bivalent *S*. *aureus* type 5 and 8 capsular polysaccharides (T5/T8) conjugate vaccine, failed to demonstrate vaccine efficacy in preventing *S*. *aureus* bacteraemia in end-stage renal disease patients receiving hemodialysis^4^. The V710 vaccine produced by Merck, that is a single conserved protein antigen IsdB, has failed in Phase III and II/III clinical trials^5^. Although a correlate of protection has not yet been established for a vaccine against *S*. *aureus* infection, one of the reasons for these failures is that we need to deepen and improve understanding of the mechanism of humoral immunity and protective cellular immune response to *S*. *aureus* infection.

There is evidence that both humoral and cellular immunity are important in preventing staphylococcal diseases^6,7^, and recent research suggests that CD4^+^ T cells play an important role in clearing pathogenic bacteria. For example, immunocompromised individuals with reduced ability to produce functional antibodies, such as those with acquired immune deficiency syndrome^8^ or defects in immunoglobulin production, have increased susceptibility to staphylococcal infections^9^. On the other hand, the help provided by CD4^+^ T cells is required to develop functional antibody responses. Moreover, cytokines secreted by T helper cells like IFN-γ and IL-17 enhance recruitment and activation of neutrophils and macrophages that provide the primary cellular defence against *S*. *aureus* infection, and several groups have demonstrated that protection induced by vaccine candidates is mediated by these two cytokines in mice^10–12^.

*S*. *aureus* manganese transport protein C (MntC) has a site that can directly combine with Mn^2+^ to bind manganese, and in animal models of *S*. *aureus* systemic infection, MntC has been found to be highly expressed on the surface of the cell membrane, and is at least partially responsible for the organism’s resistance to oxidative stress^13–18^. Furthermore, MntC has been proven to be conserved across the staphylococcal species group, and thus could confer protection against *S*. *aureus* and *S*. *epidermidis*. *In vivo* analysis of *S*. *aureus* MntC expression revealed that expression occurs very early during the infectious cycle^19^. Incontrast to MntC, neither IsdA nor IsdB protein was required for early infection events but both were required for events occurring later in infection, such as abscess formation^20^. Active immunization with MntC was effective at reducing the bacterial load associated with *S*. *aureus* and *S*. *epidermidis* infection in an acute murine bacteraemia model, and anti-MntC monoclonal antibodies exhibited a protective immunity in an infant rat passive protection model and induced neutrophil respiratory burst activity^19^. Moreover, three key effective B-cell immunodominant epitopes have been further identified on the surface of the MntC antigen, and the epitope vaccine composed of the three epitopes induces a high antibody level and provides effective immune protection and strong opsonophagocytic killing activity *in vitro* against MRSA infection^21^. However, CD4^+^ T cell-mediated immunoprotection induced by MntC of *S*. *aureus* remains unclear.

In order to investigate the role of CD4^+^ T cells induced by MntC in immune protection against *S*. *aureus* infection, we evaluated not only did MntC-specific antibodies improve survival among *S*. *aureus* peritonitis mice, but also MntC-specific CD4^+^ T cellular immune responses in which Th17/IL-17 played an important part in defence against *S*. *aureus* infection.

## Materials and Methods

### Animals and bacterial strains

Specific-pathogen-free (SPF) 6 to 8-week-old female BALB/c mice were purchased from Changchun Institute of Biological Products (Changchun, China). All experiments were approved by the Animal Ethics Committee of HeiLongJiang BaYi Agricultural University (Daqing, China) and performed in accordance with relevant guidelines and regulations.

Bacteria used in this study were as follows: *S*. *aureus* Newman, *S*. *aureus* Wood46 and *S*. *aureus* HLJ 23-1. *S*. *aureus* Newman strain^22^ (GenBank: AP009351) was kindly provided by the Eijkman Winkler Laboratory of University Medical Centre Utrecht (Utrecht, Netherlands), the Wood46 strain was maintained in our laboratory, and the HLJ 23-1 strain was isolated from milk samples of clinical bovine mastitis cases from dairy farms, and identified as *S*. *aureus* serotype 8 capsular polysaccharide by conventional microbiological methods. The bacteria were grown on tryptic soy agar (TSA) or tryptic soy broth (TSB) at 37 °C overnight. For use in experiments, bacteria were thawed, pelleted, and resuspended in the appropriate buffer or medium. Bacterial CFU were quantitated by serial dilution and plating on TSA.

### Generation of recombinant MntC protein

The *mntC* gene sequence was retrieved from the GenBank database, and was amplified by polymerase chain reaction (PCR) from genomic DNA of *Staphylococcus aureus* Newman using the primers: F: 5′-CGCGGATCCACTGGTGGTAAACAAAGCAGTGATA-3′/R: 5′-CCCAAGCTTTTATTTCATGCTTCCGTGTACAGTT-3′. The PCR products were cloned into the pMD18T-Easy vector and transformed into *Escherichia coli* DH5α. Following digestion with the restriction enzymes BamHI and HindIII, target fragments were cloned into an expression vector pET-28a and expressed in *E*. *coli* BL21 (DE3) (Tiangen, Beijing, China). The bacteria were then treated with 0.1 mM isopropyl-β-D-1-thiogalactopyranoside (IPTG, Biosharp, Hefei, China) at 37 °C for 4 h to induce the expression of recombinant protein, and the bacterial cells were harvested by centrifugation, then they were ultrasonicated, and recombinant MntC protein was acquired as a His-tagged protein that facilitated the subsequent purification process.

The His-tagged protein was purified using His-Binding-resin (Novagen, Germany). The suspension was loaded onto a Ni^2+^-charged chelating agent. Contaminants were washed away with a solution (pH 8.8) of 50 mM Tris, 250 mM NaCl and 20 mM imidazole. The recombinant MntC protein was then eluted with a solution (pH 8.8) of 50 mM Tris, 250 mM NaCl containing increasing amounts of imidazole from 20 to 500 mM. The protein eluate was subjected to endotoxin removal by Triton X-114 phase separation, after which the concentration of endotoxin was reduced to less than 2.5 pg/μg of protein. The protein was extensively dialyzed against phosphate-buffered saline (PBS) for 24 h. Purified protein samples were analysed by 12% SDS-PAGE and western blotting, then divided into small aliquots and stored at −70 °C.

### Immunization with MntC and challenge

The mice were injected intramuscularly with 50 μg rMntC emulsified with complete Freund’s adjuvant (CFA, Sigma-Aldrich, St. Louis, MO, USA). The second immunizations were given three weeks later with the same protein in incomplete Freund’s adjuvant (IFA, Sigma-Aldrich). Isovolumetric PBS plus adjuvant immunization was treated as the negative control.

Two weeks after the last immunization, the immunized BALB/c mice in the challenge group were infected by intraperitoneal injection with *S*. *aureus* Newman (8 × 10^8^ CFUs), *S*. *aureus* Wood46 (1 × 10^8^ CFUs), or *S*. *aureus* HLJ 23-1 (5 × 10^8^ CFUs). The survival rates were monitored and recorded at daily for 14 days after challenge. For bacterial burden analyses, an infective dose (2 × 10^8^ CFUs) of *S*. *aureus* Newman was administered intraperitoneally to each mouse.

### Bacterial colonization test in the blood and organs

Venous blood samples from mice were collected under asepsis condition, reserving in heparin anticoagulant tubes. Meanwhile lungs, spleens, livers and kidneys of mice were harvested, grinded and homogenated in 2 mL bacteria-free PBS. All samples needed to diluted through multi-proportion dilution method, a small amount of diluted samples were cultured on TSA for 24 h at 37 °C to calculate the CFUs of bacterial colonization in the blood and organs.

### ELISA for specific antibodies

At 7, 14, 28 and 35 days after the first immunization, the blood samples from the tail vein of mice were collected, sera were separated and used as the primary antibody for an enzyme-linked immunosorbent assay (ELISA), with MntC as the coating antigen. Horseradish peroxidase (HRP)-conjugated goat anti-mouse IgG, anti-IgG1, anti-IgG2a, anti-IgG2b and anti-IgG3 (Promega, San Luis Obispo, CA, USA) were used as the secondary antibodies respectively. The substrate was 3,3’,5,5’- tetramethylbenzidine (Sigma-Aldrich), and the stop solution was 2 M H_2_SO_4_ in this reaction. After terminating, the titres were measured at OD450 using a microplate reader (Bio-Rad, Hercules, CA, USA).

### Opsonophagocytic killing assay

Cryopreserved *S*. *aureus Newman* was thawed, amplified and cultured in TSB overnight, bacterial CFUs were determined by OD 600. The bacteria were diluted with DMEM to the appropriate concentration after being washed three times, which was applied in this experiment.

Peritoneal macrophages were obtained after injection of 1 mL of thioglycolate into the peritoneal cavity of BALB/c mice (unvaccinated or MntC-vaccinated, five for each group) 72 h prior to macrophage collection. Mice were euthanized by cervical dislocation and 5 mL of ice-cold PBS was injected into the peritoneal cavity, followed by vigorous massage. The recovered cells were cultured in DMEM (HyClone, Logan, Utah, USA) supplemented with 20% heat-inactivated FBS (HyClone), distributed in a six-well plate (NEST, Wuxi, China) at a concentration of 5 × 10^5^ cells/mL and incubated with 5% CO_2_ for 24 h to allow for adherence. Then *S*. *aureus Newman* were diluted to 1 × 10^6^ CFUs/mL, and mixed with 10 µL of the last collected mouse sera per mL. After 1 h, the mixture was added to peritoneal macrophages and incubated at 37 °C with 5% CO_2_ for 30 min. The supernatant was discarded, the macrophages were washed three times with fresh medium. To burst the macrophages, 1 mL ice cold dd-water were added the samples, the lysate was incubated at 4 °C for 30 min. After centrifuging the lysate, the supernatant was discarded, and sediment was resuspend with 0.5 mL agglutination lysis buffer (0.5% saponin, 200 U streptokinase, 100 µg trypsin, 2 µg DNase, 10 µg RNase per ml PBS)^23^. Treatment with agglutination lysis buffer for 10 min at 37 °C before culturing on TSA for enumeration of CFUs, each of sample was performed a parallel-controlled trial. The phagocytosis efficacy was defined as an increase of CFUs after 24 h culturing. Sera from PBS-vaccinated and unvaccinated mice was used as controls. This experiment was repeated three times independently with similar results.

### Proliferation assay and cytokine profile analysis of CD4^+^ T cells

Under aseptic conditions, the mice were humanely sacrificed and spleens were harvested. A single-cell suspension was harvested through a 200 μm nylon membrane. After treatment with erythrocyte lysing buffer, splenocytes were washed three times with RPMI-1640 (HyClone) and incubated for 24 h at 37 °C in 5% CO_2_, then resuspended to a density of 1 × 10^8^ cells/mL in RPMI-1640 complete medium (RPMI-1640, 10% FBS, 100 U penicillin/mL, 100 U streptomycin/mL) with the addition of 10 mg/mL mitomycin-C (Sigma-Aldrich), followed by washing with RPMI-1640 three times. The antigen-presenting cells were centrifuged, the supernatant was discarded and cells were collected and diluted to 1 × 10^5^ cells/mL. Specific splenocytes obtained from the MntC-immunized mice were harvested 7 days after the last immunization. In order to further isolate specific CD4^+^ T cells, we used OctoMACS™ immunomagnetic beads (Miltenyi Biotec, Germany) to collect CD4^+^ T cells and after diluting the cell suspension to 5 × 10^5^ cells/mL, the purity of isolated CD4^+^ T cells was analysed by flow cytometry (Supplementary Figure S2).

For the specific analysis of CD4^+^ T cell proliferation, 100 μL CD4^+^ T cells (5 × 10^5^ cells/well) and APCs (1 × 10^5^ cells/well) were seeded into 96-well flat bottom culture plates (NEST) with incomplete medium containing 1 mg of MntC at 37 °C in 5% CO_2_. ConA-stimulated cells were used as positive control, and PBS-stimulated cells were used as negative control. After 1 day of *ex vivo* antigen stimulation, cell proliferation was measured using a cell counting kit-8 (cck-8, Beyotime, Shanghai, China) according to the manufacturer’s instructions, by adding 10 μL of cck-8 to the culture medium and incubating for an additional 3 h. The absorbance was then determined at 450 nm wavelength using an ELISA Reader (Bio-Rad).

In order to determine the levels of IL-17, IFN-γ and IL-4 produced by CD4^+^ T lymphocytes, cells culture supernatants were collected and measured using commercial ELISA kits (Dakewei, Beijing, China), according to the manufacturer’s instructions.

### Specific T cell bulk culture

Spleens were harvested from the mice 7 days after the last immunization, and lymphocytes were isolated using mouse lymphocyte-separation reagent (Dakewei). Next, isolated lymphocytes were pulsed with MntC protein (0.5 µM) or peptides (5 µM) and stimulated with 5 U/mL IL-2 (PeproTech, Rocky Hill, NJ, USA) in RPMI-1640 complete medium. Half of the medium was removed when it turned yellow then replaced with fresh RPMI-1640. The lymphocytes were collected and cultured in RPMI-1640 containing 20 U/mL IL-2 on day 5. Lymphocytes were harvested and analysed at specific times.

### Determination of the percentage of Th1/Th17 cells by flow cytometry

Cultured lymphocytes were harvested and labelled CD4^+^ T cells separated by OctoMACS™. CD4^+^ T cells were incubated with 5 µM MntC in RPMI-1640 for 6 h in the presence of PMA (50 ng/mL, Sigma-Aldrich), ionomycin (1 mM, Sigma-Aldrich), and Golgistop (1 µL per 1.5 mL cell culture medium, BD Pharmingen, San Diego, CA, USA) Then the cells were washed and labelled with anti-IFN-γ-PE (eBioscience, San Diego, CA, USA), anti-IL-4-PE (eBioscience) and anti-IL-17A-FITC (eBioscience) in 1 × Permeabilization Buffer (eBioscience). Approximately 1 × 10^5^ cells were acquired using the flow cytometer CytoFLEX (A00-1-1102, BECKMAN COULTER), and the data were analysed using CytExpert software.

### Passive immunization

One day before infection, each mouse was intraperitoneally injected with 300 μL MntC-specific sera, with PBS used as negative control. Aliquots containing 1 × 10^6^ MntC-specific CD4^+^ T cells per mouse were adoptively transferred via the intravenous route, with PBS as negative control. Survival was monitored for 14 days. Bacterial burdens were analysed 24 h after infection.

### Antibody neutralization test

The MntC-vaccinated mice were treated with commercialized monoclonal antibodies to block immune function of IFN-γ or/and IL-17 *in vivo*. We could not sure that IFN-γ and IL-17 function in combination or not, so two single blocking groups and a combinative blocking group were set up respectively. The functional grade purified anti-mouse IFN-γ (aIFN-γ, eBioscience) or anti-mouse IL-17 (aIL-17, eBioscience) was diluted with PBS to 1 μg/μL to reserve. MntC-vaccinated mouse were injected intraperitoneally with 100 μg aIFN-γ, aIL-17, or both (200 μL per mouse). In addition, MntC-vaccinated mice and non-vaccinated mice were also injected with 100 μg of the nonspecific isotype control IgG in the same way, as positive and negative control group respectively. After treating 48 h, mice were challenged with *S*. *aureus* Newman (2 × 10^8^ CFUs) for bacterial colonization test in organs.

### Adoptive transfer of Th1 and Th17 cells

MntC-specific Th1 (or Th17) cells were isolated using the Mouse IFN-γ Secretion Assay Cell Enrichment and Detection Kit (or Mouse IL-17A Secretion Assay Cell Enrichment and Detection Kit) (Miltenyi Biotec) according to the manufacturer’s instructions. Briefly, splenic lymphocytes of MntC immunized mice were bulk cultured *in vitro*. Cells were then harvested and CD4^+^ T cells were enriched using commercial MACS beads designed to negatively select CD4^+^ T cells (Miltenyi Biotec). The isolated CD4^+^ T cells were stimulated with MntC for 5 h. Cells were then labelled with Mouse IFN-γ Catch Reagent (or IL-17A Catch Reagent), which could attach to the cell surface. Subsequently, the cells were incubated briefly at 37 °C to allow for cytokine secretion. The secreted IFN-γ (or IL-17A) bound to the IFN-γ Catch Reagent (or IL-17A Catch Reagent) on positive, secreting cells. These cells were then labelled with a second IFN-γ specific antibody (or IL-17A specific antibody) conjugated to phycoerythrin (PE). Finally, the IFN-γ- (or IL-17A)-secreting cells were magnetically labelled with anti-PE microBeads and enriched over a MACS column. Specific lymphocytes were collected, centrifuged, resuspended in normal saline, and injected intravenously into mice (1 × 10^6^ cells per mouse in 200 μL of PBS). The mice were challenged with *S*. *aureus* Newman on the following day.

### Statistical analysis

Data are expressed as the mean ± SEM and compared using a two-tailed Student’s t-test. The results were analysed using OriginPro 8.0 and SPSS software 12.0, and were considered statistically significant if *P* values were less than 0.05, 0.01 or 0.001.

## Results

### Expression and purification of MntC

Recombinant protein was expressed in *E*. *coli* BL21 (DE3) in the soluble fraction under the induction of 0.1 mM IPTG. We expressed the complete MntC protein, and the recombinant proteins corresponded to their predicted molecular masses. The protein was purified by His-Binding-resin, and migrated as a single major band at an apparent molecular weight of 34 kD, indicating that most of the contaminants had been removed (Supplementary Figure S1).

### MntC confers protection against *S*. *aureus* infection in the peritonitis mouse challenge model

**Figure 1:**
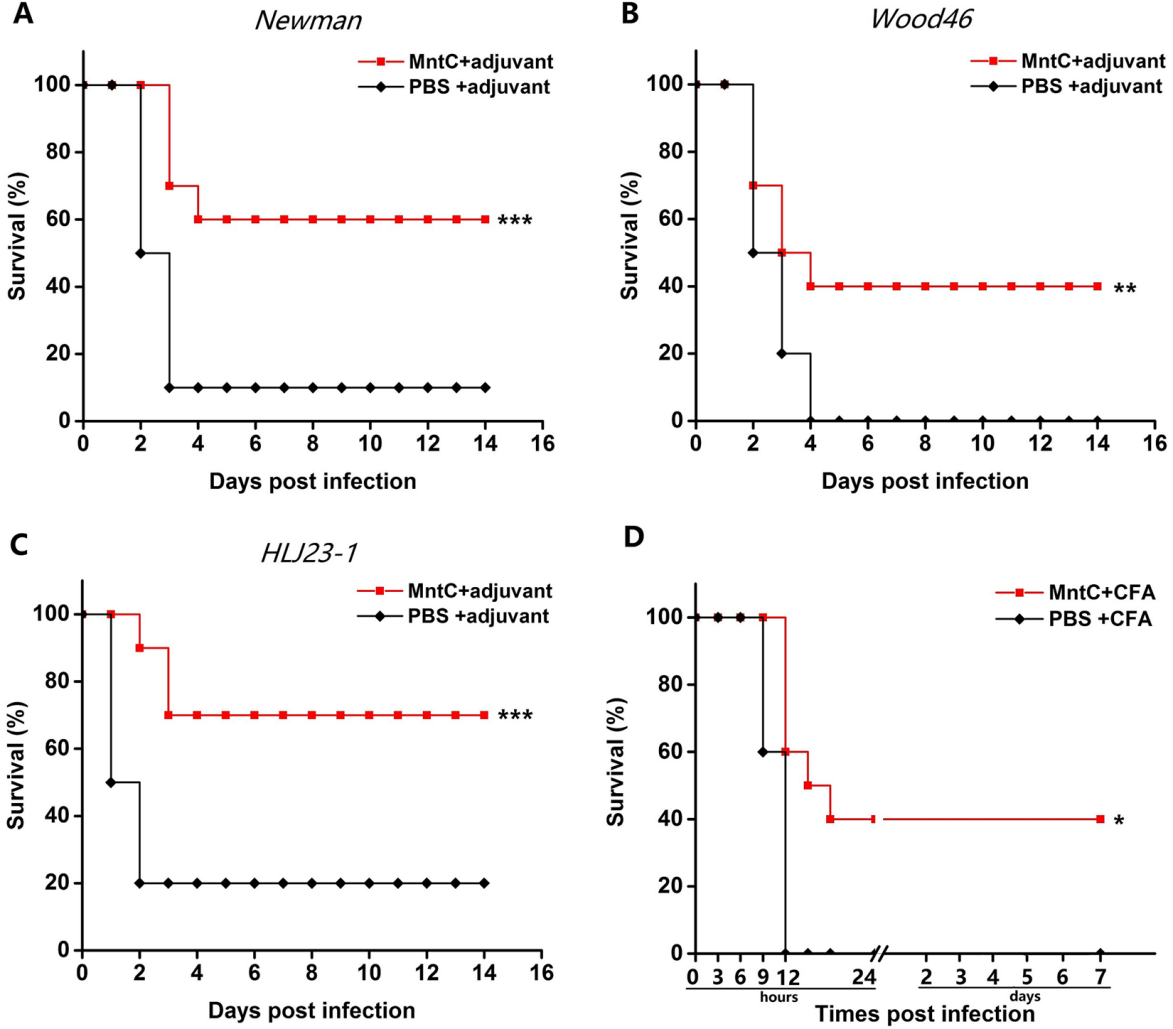
Validation of the protective efficacities in a lethal doses *S*. *aureus* peritonitis model. Female BALB/c mice (n = 10) were peritoneally challenged with (**A**) *S*. *aureus* Newman (8 × 10^8^ CFUs), (**B**) *S*. *aureus* Wood46 (1 × 10^8^ CFUs) and (**C**) *S*. *aureus* HLJ 23-1 (5 × 10^8^ CFUs) after immunization. (**D**) Immunotherapy, MntC-vaccinated mice 1 h after *S*. *aureus* Newman (8 × 10^8^ CFUs) infection. Survival rates were daily monitored and recorder for 14 days, and compared to animals treated with PBS. Significant differences were indicated by (*P < 0.05), (**P < 0.01) and (***P < 0.001).

The efficacy of MntC was evaluated in the peritonitis model by immunizing mice with adjuvant, PBS with adjuvant was used as control. Two weeks after immunization, mice were infected i.p. with S. aureus Newman (8 × 10^8^ CFUs), HLJ 23-1 (5 × 10^8^ CFUs) or Wood46 (1 × 10^8^ CFUs), and then monitored for up to 14 days. MntC-immunized mice showed substantially improved survival rates 7 days post-challenge compared to adjuvant control (Fig. 1A–C). As expected, an increased survival against *S*. *aureus* challenge was measured after booster immunization with MntC plus adjuvant, and showed statistically significant differences compared with control mice immunized with PBS plus adjuvant. These data indicated that MntC confers protection against infections of different *S*. *aureus* strains.

We also found that immunization with MntC/adjuvant 1 h after i.p. challenge with *S*. *aureus* Newman (8 × 10^8^ CFUs) similarly confers protection (Fig. 1D). Therefore, as an effective *S*. *aureus* vaccine antigen, MntC was demonstrated to be a potentially valuable therapeutic target. However it remains unclear how to induce protection by MntC.

### Active immunization with MntC induces a strong antibody response

**Figure 2:**
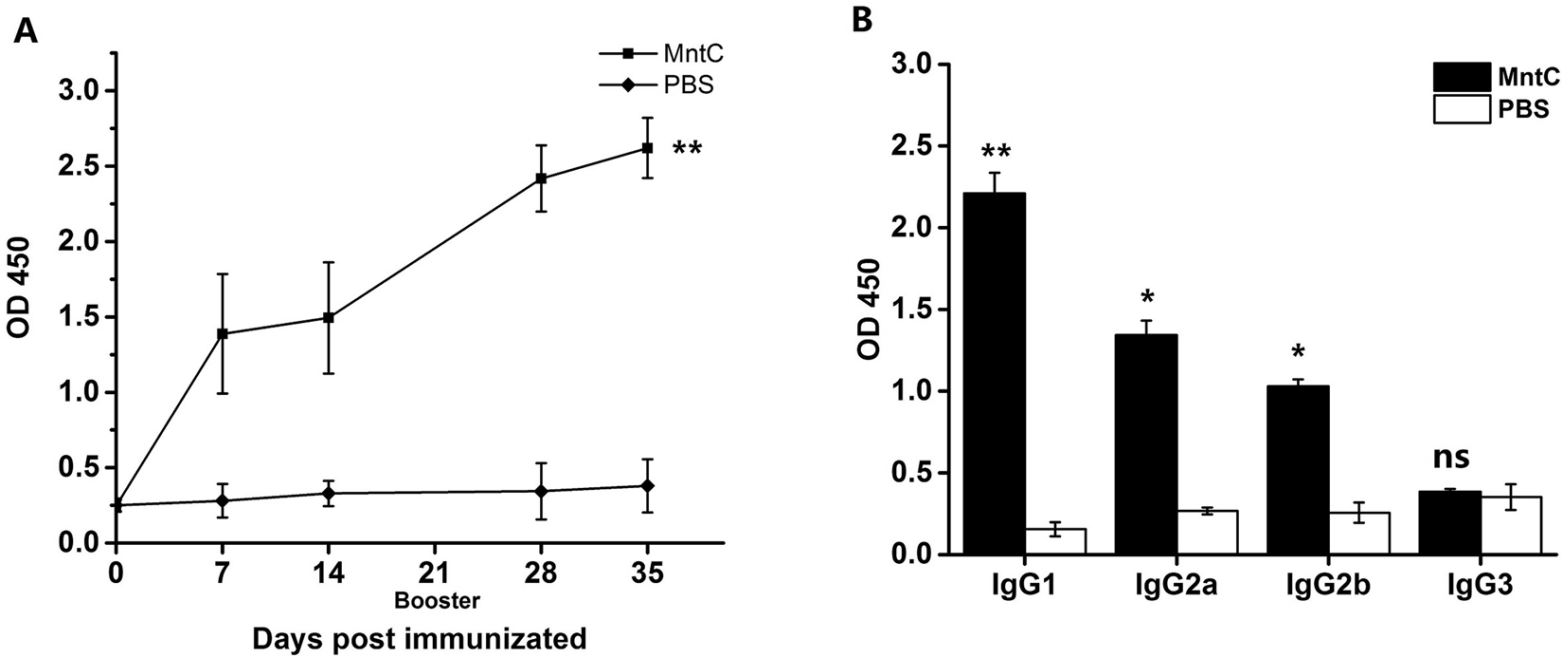
Active immunization with recombinant MntC induced specific IgG production. Female BALB/c mice (n = 4) were intramuscularly immunized with purified MntC. Seven days after the booster immunization, the mice were bled, and the sera were tested by ELISA for anti-recombinant protein antibodies. The titres were determined for (**A**) IgG, (**B**) IgG1, IgG2b, and IgG3. The results represent the mean and standard error of each group of mice. Significant differences were indicated by (*P < 0.05), (**P < 0.01) and (***P < 0.001).

We analysed the total IgG titer in the sera from MntC-vaccinated mice, and found that MntC could induce a strong humoral immune response after the intensify immunization (Fig. 2A). Moreover, the IgG subclass induced by MntC was analysed in the same way, IgG2a, IgG2b as well as IgG1 titer in MntC-specific sera was considerably higher than that in the control sera (Fig. 2B). It is well-known that markers of IgG2a and IgG1 represent Th1 and Th2 cell responses, respectively. Therefore we suggested that both Th1 and Th2-biased responses had been induced.

### MntC-specific antibodies possess better opsonophagocytic killing activity *in vitro*

**Figure 3:**
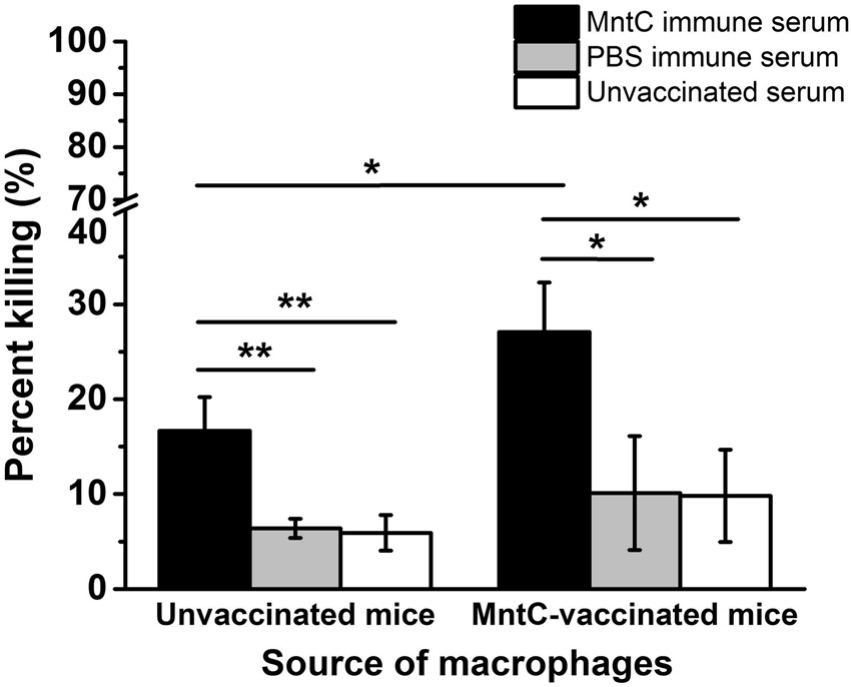
Opsonophagocytic activity mediated by immune sera from mice immunized with MntC. BALB/c mice (n = 5) were immunized with MntC or PBS, sera was separated. Source of peritoneal macrophages was MntC-vaccinated mice and unvaccinated mice respectively. 10 µL immune sera, 5 × 10^5^ mouse peritoneal macrophages and 1 × 10^6^ CFUs *S*. *aureus* Newman were incubated in round-bottomed six-well plates for 30 min before treating with agglutination lysis buffer and plating on TSA for CFUs. Then the percentage of phagocytosis was counted. PBS-vaccinated sera were used as negative control, and untreated sera were used as blank control. Significant differences were indicated by (*P < 0.05), (**P < 0.01) and (***P < 0.001).

Vaccine phagocytosis and processing to present antigens has been shown to be an important factor responsible for the capacity to induce a protective immune response. Although immunization with MntC can induce a strong humoral immune response *in vivo*, whether these antibodies fulfilled an antibacterial function *in vitro* remained unclear. To determine the activities of MntC-specific antibodies, an opsonophagocytic killing assay (OPKA) which measured antibody-mediated microbirorous ability *in vitro* was carried out. As shown in Fig. 3, peritoneal macrophages from unvaccinated mice were used for assay, OPK activities of MntC-specific sera, adjuvant control and blank control were 16.67%, 6.39%, and 6.33%, respectively. These findings manifested that the MntC-specific antibodies were more contributed to cytophagy capacity of macrophages against *S*. *aureus*. Furthermore, macrophages from MntC-vaccinated mice as phagocytes, OPK activities of MntC-specific sera group were enhanced to 27.07%. Results significant difference between the two groups of macrophages (P < 0.05), with statistical significance. Thus, active immunization with recombinant MntC promoted the phagocytosis of macrophages.

### Proliferation response and cytokine production of CD4^+^ T cells

**Figure 4:**
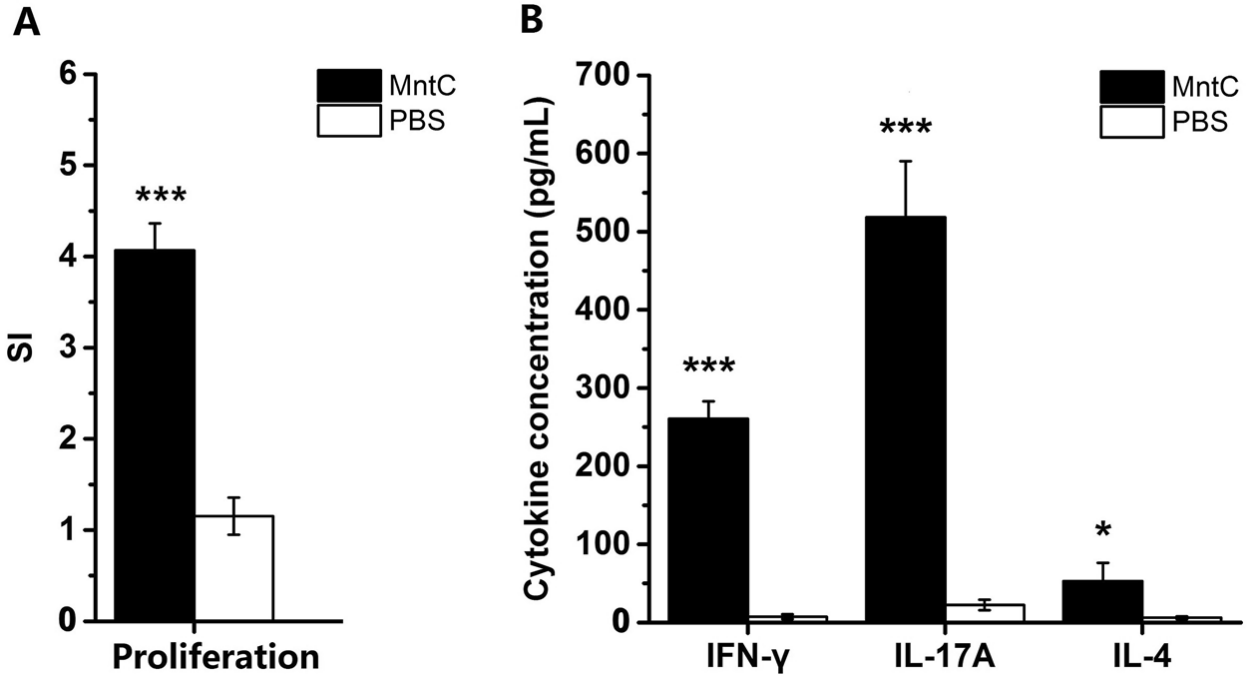
Proliferative responses and cytokine secretion of MntC-vaccinated CD4^+^ T cells in mice. (**A**) Proliferative response assay. One week after the last immunization with MntC or PBS, spleen lymphocytes were isolated from BALB/c mice (n = 5), and CD4^+^ T cells were incubated with 1 mg synthetic peptides plus mitomycin C-treated naïve syngeneic feeder cells for 1 day, at a ratio of 5: 1 × 10^5^ cells. A significant difference was observed, which was in comparison with the PBS negative control group. (**B**) Secretion levels of IFN-γ, IL-17A and IL-4 of CD4^+^ T cells were determined by ELISA. The solid bars represent the mean values ± standard deviation (SD). Significant differences were indicated by (*P < 0.05), (**P < 0.01) and (***P < 0.001).

We evaluated MntC-specific cellular responses. CD4^+^ T cells from MntC-vaccinated mice showed selective significant proliferation compared to control group (Fig. 4A). Next, level of cytokines produced by CD4^+^ T cells was measured. The results showed that level of IFN-γ, IL-17A and IL-4 that secreted by MntC-specific CD4^+^ T cells, which increased significantly relative to PBS control group, particularly secretion of IL-17A (Fig. 4B). We deduced that CD4^+^ T cells were stimulated and polarized toward Th1, Th17 and Th2 sub-group subsets.

### Analyses of percentage of MntC-specific Th1, Th2 and Th17 cells by flow cytometry

**Figure 5:**
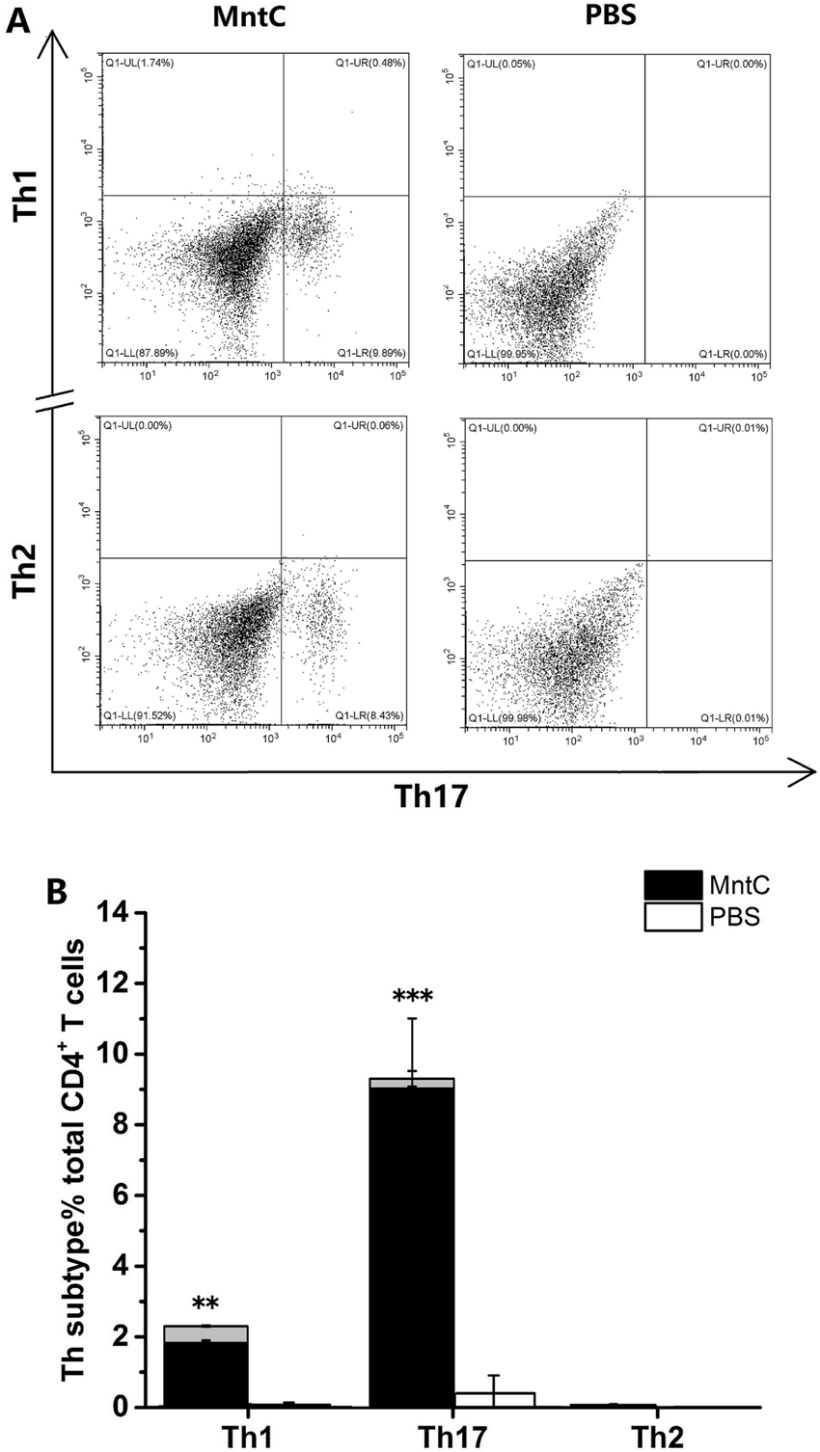
Phenotypic analysis of CD4^+^ T cells after immunization by MntC. (**A**) After 7 days’ incubation, lymphocytes were collected and treated with PMA, ionomycin and Golgistop for 6 hours. Then these cells were washed and stained with fluorescent antibody for Th1, Th17 and Th2, analyzing by flow cytometry. (**B**) Th1, Th17 and Th2 cell responses of all immunized mice were statistically analysed. The grey bars represent the proportion of double-positive cells. Significant differences were indicated by (*P < 0.05), (**P < 0.01) and (***P < 0.001).

MntC-specific lymphocytes were harvested after culture for 7 days for CD4^+^ T cell subtyping *in vitro*. After stimulation, CD4^+^ T cell in MntC group more polarized to Th17 cells and slightly polarized to Th1 cells (Fig. 5). At the same time, very few Th1/Th17 and Th2/Th17 double-positive cells were detected. There are no polarization in PBS negative control group.

### Passive immunization decreased bacterial colonizations in the blood and organs to increase survival rate

Three hundred microliters of sera from MntC-vaccinated mice were injected into the peritoneal cavity of naïve mice. Meanwhile 1 × 10^6^ CD4^+^ T cells were also injected intravenously into naïve mice, with PBS used as negative control. After 24 h mice were injected with a non-lethal dose of *S*. *aureus* Newman for analysis of bacterial burdens and the minimum lethal dose for survival was analysed.

**Figure 6:**
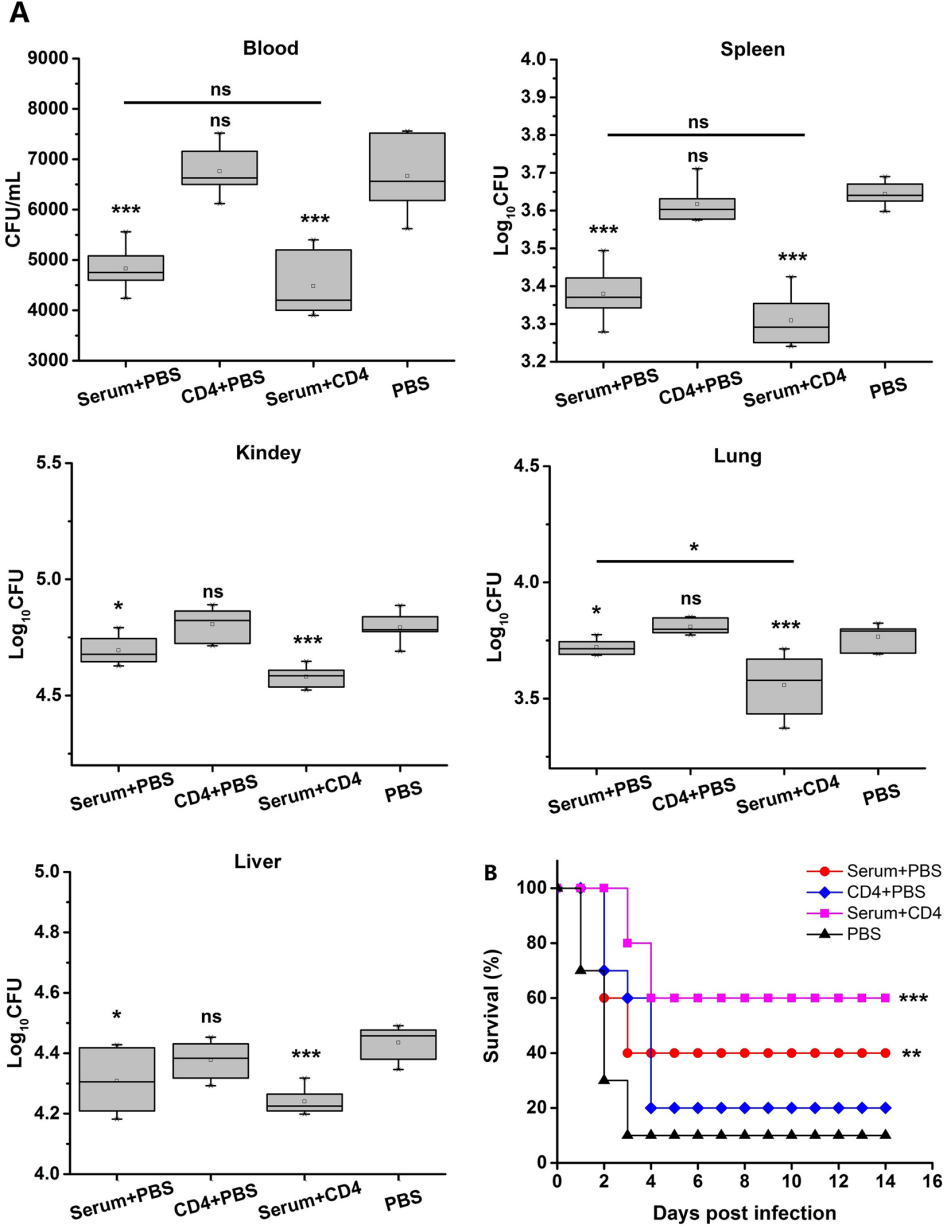
Passive immunization used MntC-specific CD4^+^ T cells (and sera). One day before infection, 300 µL MntC-specific sera or (and) 1 × 10^6^ CD4^+^ T cells per mouse were passively immunized via the intraperitoneal route, with isometric PBS as negative control. (**A**) Quantitative bacteriology was performed in organs. (**B**) Survival was monitored for 14 days. Significant differences were indicated by (*P < 0.05), (**P < 0.01) and (***P < 0.001).

The bacterial colonizations in the blood, spleens, kidneys, lungs and livers were computed 24 h post-infection. In the blood, the bacterial colonizations in the sera and CD4^+^ T cell group were considerably reduced compared to the control group, and the single sera group also showed a statistically-significant reduction, but this immunity effect was lower than in the sera and CD4^+^ T cell group. The single CD4^+^ T cell group did not show a significant difference. Similarly in the spleens, kidneys, lungs and livers, the ability of removing *S*. *aureus* was improved in the sera of the group treated with CD4^+^ T cells and the single sera group. Consistent with the bacterial burden, passive immunization with sera with CD4^+^ T cells induced the highest survival rates, and sera alone induced good protection, while in the group treated with CD4^+^ T cells alone the death of infected mice was delayed to a certain degree (Fig. 6). These data showed that MntC-specific antibodies provided effective protective barriers for *S*. *aureus* infection via eliminating *S*. *aureus* colonization in the blood and organs. These also demonstrated that CD4^+^ T cells play an indirect role in protection that is important and necessary.

### IL-17 secreted by Th17 cells plays an indispensable part in protection efficacy

**Figure 7:**
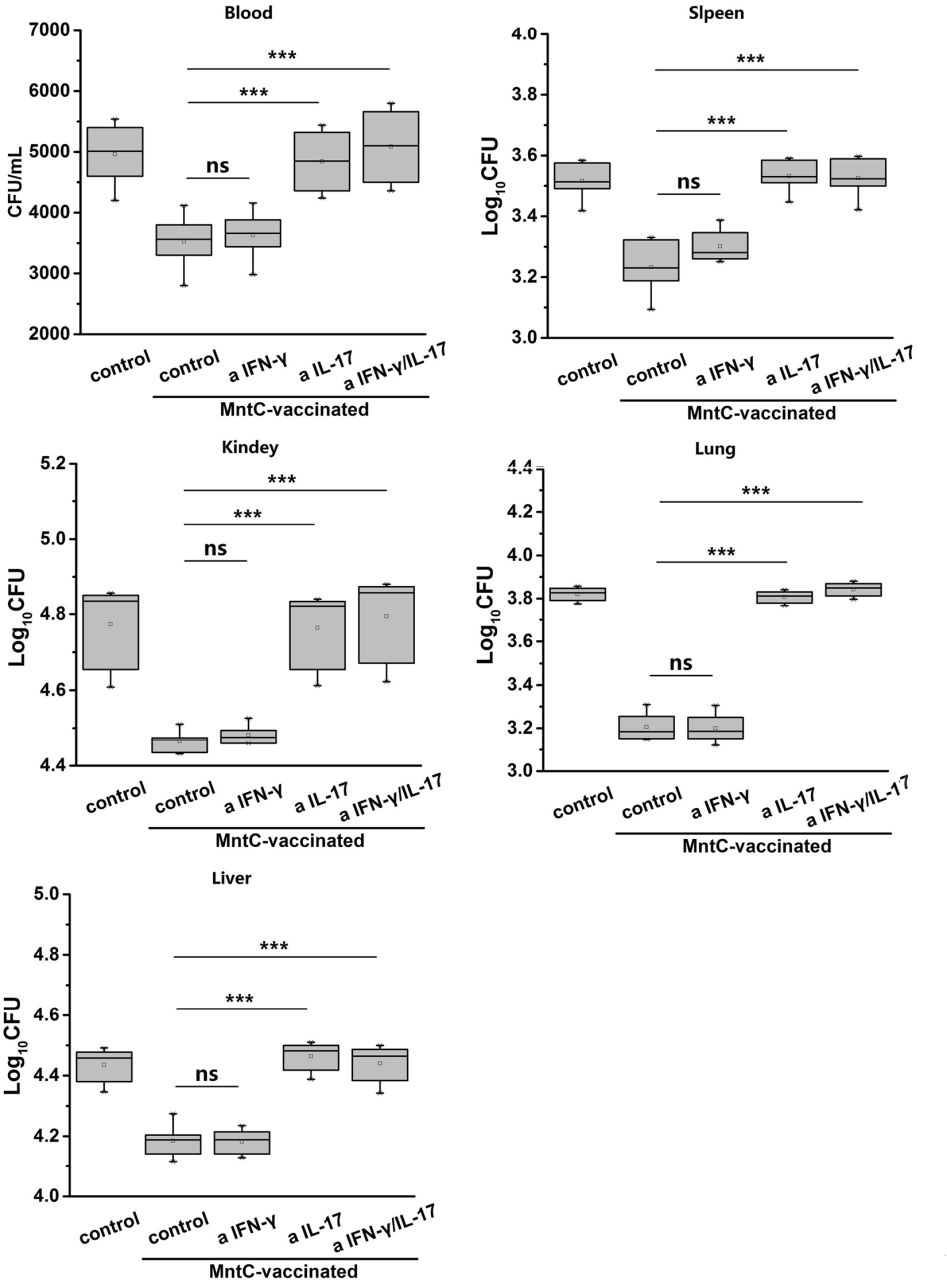
Neutralization of IL-17 resulted in a apparent increase of bacterial colonization *in vivo*. MntC-vaccinated mice (n = 5) were intraperitoneally injected with anti-IFN-γ or (and) anti-IL-17, infected non-lethal dose *S*. *aureus* Newman 2 days later. One day post-infection, the mice were sacrificed, their (**A**) blood, (**B**) spleens, (**C**) kidneys, (**D**) lungs and (**E**) livers were harvested and homogenated to measure the CFUs of bacterial colonization. Mice immunized with MntC did not obtained a apparent protection to eliminate bacteria in mice, when the biological activities of IL-17 was inhibited. The CFUs of bacterial colonization in non-vaccinated mice and vaccinated without neutralizing cytokines mice were considered as the control groups, respectively. Significant differences were indicated by (*P < 0.05), (**P < 0.01) and (***P < 0.001).

In order to authenticate immune function of IFN-γ and IL-17 in the protection, we injected with neutralizing-antibodies to MntC-vaccinated mice for blocking their cytokines. After neutralizing 48 h, mice was infected with *S*.*aureus*, then bacterial colonizations in the blood and organs were measured 24 h post-infection. In the non-neutralizing mice, bacterial colonization in MntC-vaccinated mice was substantially lower than non-vaccinated mice. When IFN- was neutralized alone, the bacterial colonization in the blood and organs did not change significantly compared with the non-neutralizing vaccinated mice, illustrating neutralization of IFN-γ had no impact on protection. Nevertheless, when IL-17 was blocked, bacterial burden in mice had a significant increase, it was closed to non-vaccinated mice. Neutralization of IFN-γ and IL-17 combined similarly led to deletion of protection (Fig. 7).

**Figure 8:**
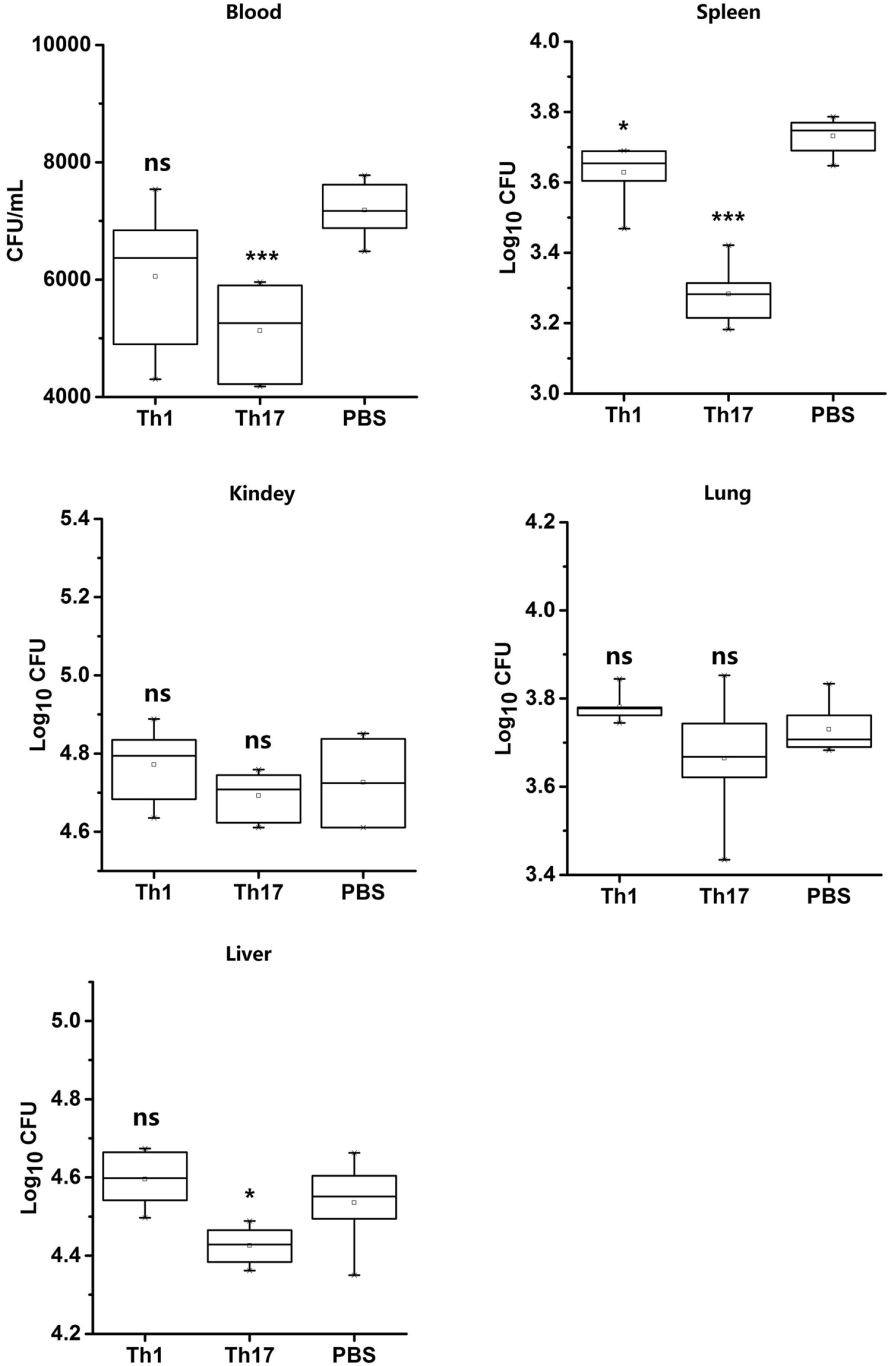
Th17 cells were required for MntC protection. BALB/c mice (n = 3) were intraperitoneal infected with *S*. *aureus* Newman (2 × 10^8^ CFUs) after adoptive transfer (i.v.) of 1 × 10^6^ MntC-specific Th1 or Th17 cells. Calculation method of bacterial colonization in the blood and organs was consistent as described above. Asterisks indicate significant differences between vaccinated and control mice. Significant differences were indicated by (*P < 0.05), (**P < 0.01) and (***P < 0.001).

Although we found that the numbers of Th1 cells were lower than Th17 cells, we did not know whether or not the same amounts of Th1 cells would increase protection. Consequently we designed an adoptive transfer test to confirm the results. Our results shown that the bacterial colonization in the Th17 cells-receptor mice was markedly decreased compared with negative control mice. In contrast, the bacterial burdens in the Th1 cells-receptor mice were lower than in the control group, but this difference did not reach statistical significance (Fig. 8). We thus considered that IL-17 secreted by Th17 cells plays an indispensable part in protection efficacy.

## Discussion

Although *S*. *aureus* colonization cannot lead to infection in healthy individuals generally, it does increase the risk of harm due to infection during surgery or trauma. Vaccines are promising alternatives to serious antibiotics. However, no anti-*S*. *aureus* vaccine has yet been successful^24–26^, and one of the main reasons is that the immune mechanism of a vaccine against *S*. *aureus* infection has not been fully elucidated.

We confirmed that MntC immunization could induce mice to produce effective protection against challenge with three *S*. *aureus* strains in a murine peritonitis model. In addition, we found that mice immunized with MntC plus Freund’s adjuvant 1 h after challenge with *S*. *aureus* Newman strain still showed an effective protective response. Therefore, MntC can be used not only to prevent, but also to treat, *S*. *aureus* infection.

The humoral immune response and cellular immune response are the main components of immune protection. In this study we analysed the protective humoral and CD4^+^ T cellular immune responses, in an attempt to elucidate the roles of antibodies and cytokines in immunity protection.

There are a number of examples where antibodies have been shown to afford some protection against *S*. *aureus* infection^27–32^. Possibly these antibodies interact with the pathogen shortly after infection, and mediate antibody-dependent cell killing activities^28,33,34^. The MntC vaccine induced high levels of IgG in sera, and the IgG subtype tended to be IgG1. However, it is still unclear that whether the antibody responses mediated immune protection against *S*. *aureus* infection. Therefore we evaluated the MntC-induced humoral immune protection through opsonophagocytic killing assay and passive immunization test. The results showed that MntC-specific antiserum effectively promoted phagocytosis of *S*. *aureus* by macrophages, and the phagocytosis of the macrophages from the mice immunized with MntC was significantly better than that from untreated mice. In addition, MntC-specific antiserum obviously decreased bacterial load in organs and the death rate by challenge. These results indicated that MntC-specific polyclonal antibodies can mediate effective immune protection against *S*. *aureus* infection.

Although antibodies might contribute to protection, cellular immune responses, and especially CD4^+^ T cells, are crucial for protective immunity against intracellular pathogenic bacteria^35,36^. Previously, we successfully identified an immunodominant CD4^+^ T cell epitope of MntC that _227_KHKLKHLLVETSVDKKAMES_246_, but protective effect of MntC-specific CD4^+^ T cells has not been clarified^37^. In this experiment, the MntC-specific antiserum alone provided 30% protection against *S*. *aureus* infection compared to the control. Meanwhile antiserum together with CD4^+^ T cells provided 50% protection compared to the control. These findings suggest that CD4^+^ T cells are essential to prevent infection of *S*. *aureus*. Increasing evidences has discovered that CD4^+^ T cells, especially Th1 and Th17 cells, play an vital role in vaccine-mediated protective effects against pathogens apart from the protective effects of antibodies^11,38^. Therefore we adoptively transferred CD4^+^ T cells alone into mice, and found that the CD4^+^ T cells provided only 10% protection against *S*. *aureus* infection compared to the control and barely reduced the bacterial load. All the aforementioned results demonstrated that CD4^+^ T cells in combination with antibodies can play a better role in preventing *S*. *aureus* infection.

There are evidences that the polarized immune responses of Th1 or Th17 cells has close relationship to the activation and regulation of neutrophils and macrophages^39,40^. Th1 cells are the major effector T cell population in phagocyte-mediated host defence. The principal function of Th1 cells is in producing IFN-γ, a proinflammatory cytokine which enhances the phagocytic activities of macrophages and activates macrophages to ingest and destroy microbes. Th17 cells combat microbes by producing mainly IL-17 and IL-22, and recruiting leukocytes, mainly neutrophils, to sites of infection^41^. IL-17 is an important link between T cell-mediated adaptive immunity and the acute inflammatory response, and IL-22 serves to maintain epithelial integrity, mainly by promoting the barrier function of epithelia, stimulating repair reactions, and inducing production of anti-microbial peptides. We analysed the cytokine levels of MntC-specific CD4^+^ T cells, and found that the levels of IFN-γ and IL-17A were significantly higher than those of the control group, but MntC induced more IL-17 compared with production of IFN-γ. We further analysed the frequency of Th1 and Th17 cells using flow cytometry, since Th17 cells were confirmed to exhibit both instability (they can cease to express their signature cytokine, IL-17) and plasticity (they can start expressing cytokines typical of other lineages) upon *in vitro* re-stimulation^42,43^, we established Th1/Th17 double-positive cell groups and detected a small number of Th1/Th17 double-positive cells, and found that the numbers of Th1 cells were lower than Th17 cells. This result was consistent with that of cytokine detection. To identify the antimicrobial effects of IFN-γ and IL-17, we neutralized the cytokines with monoclonal antibodies *in vivo*. IFN-γ was not secreted only by Th1 cells and IL-17 was not secreted only by Th17 cells^40,44^, however it was inhibited that biological effects of the IFN-γ or IL-17 produced by various immune cells *in vivo*. Notably, neutralization of IL-17 alone eliminated the protective efficacy mediated by MntC, lost of activity of IFN-γ had no effect on reduction of bacterial burdens. According to the above results, we considered that in spite of MntC could induce large amounts of IFN-γ and IL-17 production in mice, the protective efficacy primarily relied on the IL-17 functional pathway.

In order to confirm that the protective effect is IL-17-dependent, rather than IFN-γ dependent, we adoptively transferred equal numbers of Th1 and Th17 cells from immunized mice into naïve mice, and evaluated the bacterial load in organs after challenge. The results showed that the bacterial load in organs of Th17 recipient mice was considerably lower than in IFN-γ recipient mice. This result was consistent with recent reports that the activated Th17 cells confer efficient protection to prevent *S*. *aureus* infection^12,41,45–48^.

Some studies reported a phenomenon that was IgG1 antibody titres do not correlate with the number of Th2 cells^49,50^, we got similar results in our work. We surmised that one aspect, early studies shown that it has been easy to overlook the production of IL-4 during infection using the classical method of lymphocyte culture with antigen and measuring cytokines in the supernatant, at least in part, to the absorption of IL-4 by activated B lymphocytes^51^. Furthermore, we did not add additional IL-4 in specific T cell bulk culture, but Th2 cells differentiation were stimulated by IL-4, so we only detected a small number of Th2/Th17 double-positive cells by flow cytometry. In other aspect, this may be caused by different strains of mice^52^.

In this study, humoral immune responses and CD4^+^ T cell-mediated immune responses induced by MntC antigen were evaluated. The results demonstrate that both antibodies and Th17/IL-17 are indispensable in protective immunity against *S*. *aureus* induced by MntC. This study provides a valuable reference for design of safer and more effective vaccines against *S*. *aureus* in future.

## Electronic supplementary material

Supplementary Figure S1. Supplementary Figure S2.
