# Supplementary material for: Protective humoral and CD4+ T cellular immune responses of Staphylococcus aureus vaccine MntC in a murine peritonitis model
Source: Sci Rep. 2018 Feb 26;8:3580. doi: 10.1038/s41598-018-22044-y (PMC5832154; doi:10.1038/s41598-018-22044-y)

Protective humoral and CD4$^+$ T cellular immune responses of *Staphylococcus aureus* vaccine MntC in a murine peritonitis model

Wei Yu, Di Yao, Simiao Yu, Xintong Wang, Xiaoting Li, Mengyao Wang, Shuo Liu, Zhenyue Feng, Xiaoting Chen, Wanyu Li, Lizi Wang, Wei Liu, Jinzhu Ma, Liquan Yu, Chunyu Tong, Baifen Song, Yudong Cui*

College of Life Science and Technology, Heilongjiang Bayi Agricultural University, Daqing, Heilongjiang Province, 163319, China

* Corresponding author Email: cuiyudong@yahoo.com

Supplementary Information

This file contains 2 supplementary figures.

Supplementary Figure S1. Cloning, expression and purification of recombinant MntC. (A) Identification of MntC-pMD18T by PCR and double enzyme digestion. Lane M: DNA Marker DL5000; Lane 1: PCR product; Lane 2: pMD18T-MntC; Lane 3: Negative control; (B) Identification of MntC-pET28a by PCR and double enzyme digestion. Lane M: DNA Marker DL8000; Lane 1: PCR product; Lane2: pET28a-MntC; Lane3: Negative control; (C) SDS-PAGE and western-blotting analysis of expressed and purified recombinant protein MntC. Lane M: PAGE Ruler Prestained Protein Ladder; Lane 1: E. coli BL21(DE3) with pET-28a before induction; Lane 2: E. coli BL21 (DE3) with pET-28a induced by IPTG; Lane 3: E. coli BL21 (DE3) with pET-28a/MntC before induction; Lane 4: E. coli BL21 (DE3) with pET-28a/MntC induced by IPTG; Lane 5: Purified MntC protein. (D) Western blotting of purified MntC, anti-His antibodies were tested for primary antibody.

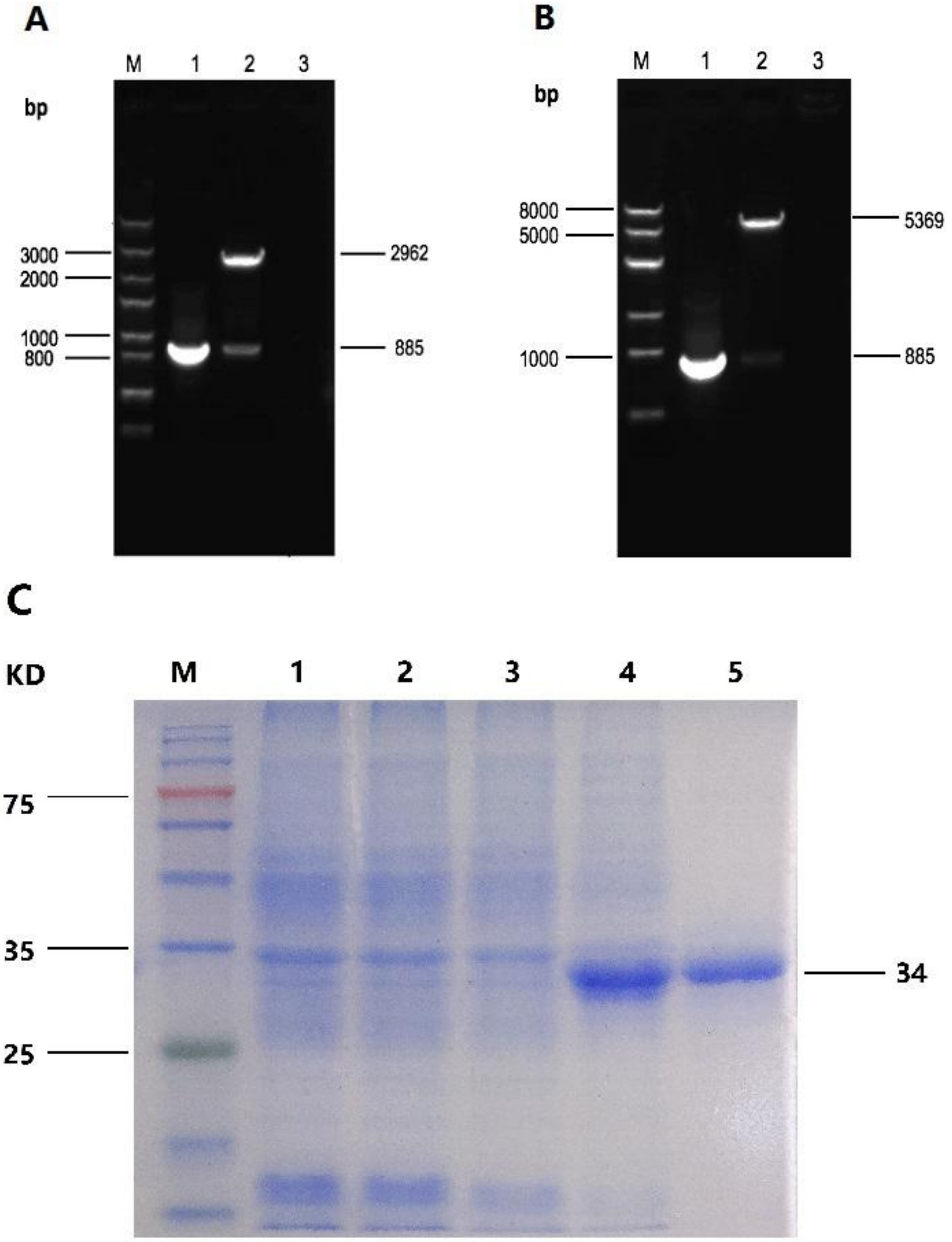

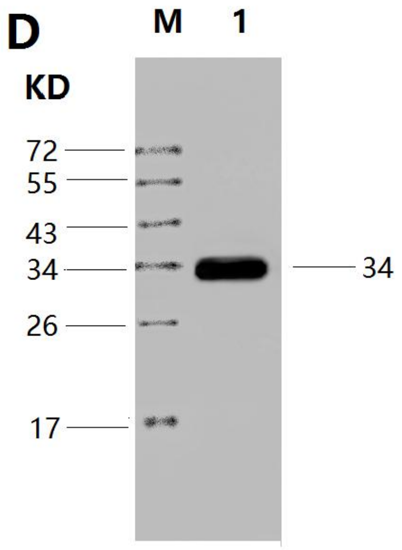

Supplementary Figure S2. Purity of isolated CD4^{+} T cells. Isolated CD4^{+} T cells treated with anti-CD4-PE demonstrated by flow cytometry analyses, the purity was greater than 95%.

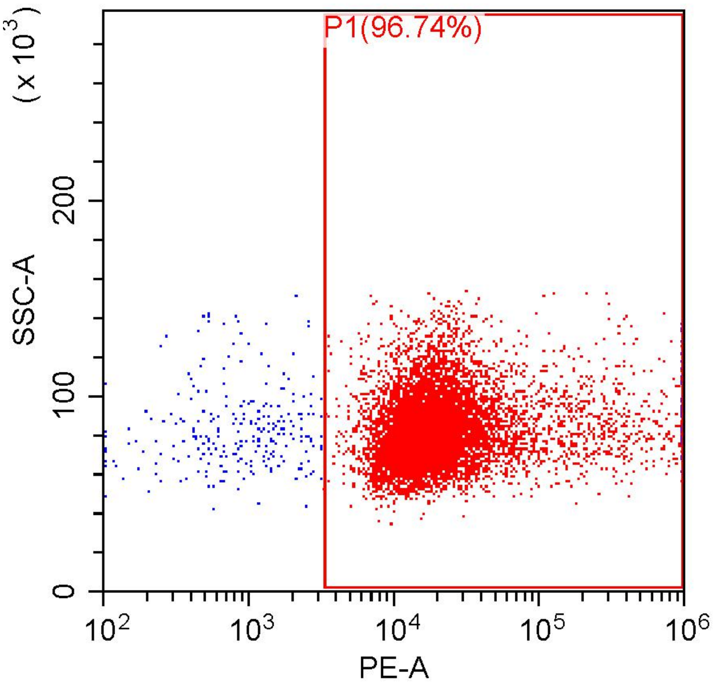

Supplement: Supplementary file 1 — Supplementary Figure S1. Supplementary Figure S2. [file 41598_2018_22044_MOESM1_ESM.pdf]
